# Pathophysiology of Fibrosis in the Vocal Fold: Current Research, Future Treatment Strategies, and Obstacles to Restoring Vocal Fold Pliability

**DOI:** 10.3390/ijms20102551

**Published:** 2019-05-24

**Authors:** Yoshihiko Kumai

**Affiliations:** Department of Otolaryngology Head and Neck Surgery, Kumamoto University School of Medicine, Kumamoto 860-8556, Japan; kumayoshi426yk@gmail.com; Tel.: +81-96-373-5255； Fax: +81-96-373-5256

**Keywords:** vocal fold, scar, tissue engineering, adipose-derived stem cell, bone marrow derived stem cell, anti-fibrotic agents, pliability, anti-inflammatory cytokine, exosome, gene therapy, laser therapy

## Abstract

Communication by voice depends on symmetrical vibrations within the vocal folds (VFs) and is indispensable for various occupations. VF scarring is one of the main reasons for permanent dysphonia and results from injury to the unique layered structure of the VFs. The increased collagen and decreased hyaluronic acid within VF scars lead to a loss of pliability of the VFs and significantly decreases their capacity to vibrate. As there is currently no definitive treatment for VF scarring, regenerative medicine and tissue engineering have become increasingly important research areas within otolaryngology. Several recent reviews have described the problem of VF scarring and various possible solutions, including tissue engineered cells and tissues, biomaterial implants, stem cells, growth factors, anti-inflammatory cytokines antifibrotic agents. Despite considerable research progress, these technical advances have not been established as routine clinical procedures. This review focuses on emerging techniques for restoring VF pliability using various approaches. We discuss our studies on interactions among adipose-derived stem/stromal cells, antifibrotic agents, and VF fibroblasts using an in vitro model. We also identify some obstacles to advances in research.

## 1. Introduction

Communication by voice depends on symmetrical vibrations within the vocal folds (VFs) and is indispensable for various occupations (e.g., teacher, doctor, and sales representative). Unexpected dysphonia may force individuals to leave their jobs and could drastically decrease their quality of life. VF scarring is one of the main reasons for permanent dysphonia and results from injury to the unique layered structure of the VFs. VF scars result in a loss of pliability and a negative alteration of viscoelasticity within the VFs and thus significantly impair VF vibration. The patient’s voice may become hoarse, and this could have a considerable impact on their quality of life. Although modern phonosurgical methods can resolve many VF pathologies, VF scarring remains clinically challenging [1,2,3,4,5].

The two major causes of VF scarring are trauma (e.g., irradiation, intubation, vocal abuse, or phonosurgery) and inflammation (e.g., laryngitis, tobacco smoking, or exposure to dust) [1,2,3,4,5]. As there is currently no definitive treatment for VF fibrosis/scarring, regenerative medicine and tissue engineering have become increasingly important research areas within otolaryngology. The main pathological features of VF scarring are disorganized composition of the extracellular matrix (ECM) and reduced pliability of the superficial layer of the lamina propria (SLP) within the VF [1,2,3,4,5]. Therefore, to successfully treat VF scarring, the pliability of the SLP and normal structure of the ECM need to be restored.

Several recent reviews have described the problem of VF scarring and various possible solutions [6,7,8,9,10,11,12,13,14,15,16], including biomaterial implants, tissue engineered cells and tissues, stem cells, growth factors and anti-inflammatory cytokines, antifibrotic agents, laser therapy and gene therapy. Despite considerable research progress, these technical advances have not been established as routine clinical procedures. This review focuses on emerging techniques for restoring VF pliability using various approaches, including in vitro and/or in vivo experimental models, regenerative medicine, tissue engineering and clinical trials. We also discuss our own studies of interactions among adipose derived stem/stromal cells (ASCs) [17,18], antifibrotic agents [19] and VF fibroblasts using an in vitro model. Additionally, we identify and discuss some obstacles to advances in research.

## 2. The Unique Microstructure of the VF and Imaging the Structure

Minoru Hirano described his innovative “body-cover model” of voice production in 1974 [20]. The VF “cover” consists of the epithelium, basement membrane zone [21] and SLP layer. These behave collectively as one functional unit. Collagen anchoring fibers in the basement membrane zone link the basal cells with the SLP [21]. The VF “body” consists of the intermediate and deep layers of the lamina propria (LP), which is firmly attached to the vocalis muscle; these two layers form the vocal ligament [21]. The vocal ligament protects the SLP from excessive stress when high-frequency sound is produced [22,23]. The key to healthy VF vibration is the pliability of the SLP. This specialized layer is typically 2 mm thick in humans and consists of highly pliable connective tissue that vibrates during phonation [24]. The ECM contains hyaluronic acid (HA), different types of collagen, and fibronectin. The SLP is soft [25] and contains fine elastin and collagen fibers embedded in mixture of proteoglycans which are relatively sparse compared to deep layers [26] and HA. Gray et al. used VFs from human cadavers to show that HA was the key for the maintenance of the low viscosity of the SLP. In contrast, the vocal ligament contains significantly more collagen and elastin fibers [25,26]. 

From the clinical point of view, accurate evaluation of the unique microstructure of the normal and scarred VF in patients is substantially significant. Burns reviewed previous studies related to the innovative technique, optical coherence tomography to image the larynx during diagnosis and treatment of various laryngeal disorders. He mentioned in the paper that precise delineation of VF-layered microstructure provides useful information for precise detection of the VF lesions along the unique layered structure [27]. Recent studies [28,29,30] presented the usefulness of this technique especially during surgical intervention to the various types of the VF lesions including VF scars. Heris et al. recently characterized the VFs microstructure and elasticity using nonlinear laser scanning microscopy and atomic force microscopy-based indentation, respectively [31]. Herrera et al. [32] and Kishimoto et al. [33] described the feasibility of magnetic resonance imaging (MRI) for resolving anatomic substructures within naïve VF mucosa, qualitative and quantitative features of acute injury, the presence of chronic scars and localization of the implanted materials. These new technologies would make it possible to evaluate the pre- and post-state of the VFs microstructure in VF scar patients.

## 3. Histopathology of VF Scarring In Vivo and In Vitro

In response to damage, the VF initiates events that are similar to those that occur when the skin is wounded. These events are for closing the wound with reconstruction of the subepithelial connective tissue so that it can resist mechanical stresses [34]. Hirano et al. examined the histopathology of VF scars from early glottic cancer patients who underwent endoscopic cordectomy, and one patient who underwent superficial cordotomy for idiopathic scarring. Patients who had undergone deep resection of the LP had excessive levels of disorganized collagen fibers and decreased levels of decorin, whereas patients who had undergone more superficial resections had fewer, better organized collagen fibers. In addition, elastin, HA, and fibronectin levels varied significantly among the patients, regardless of the depth of resection [35].

Welham et al. [36] previously reviewed various VF scarring animal models in detail, providing a basis for the development of antifibrotic therapies [37,38,39,40,41,42,43,44,45,46,47,48,49,50,51,52,53,54,55]. Obviously, animal models can be used to systematically address scarring issues that cannot be examined in patients. For VF scarring, important characteristics include the size, shape and structure of the scar, presence or absence of the vocal ligament, and the organization of ECM components. These studies were consistent with observations in patients and supported the conclusion that excessive collagen (especially type I collagen) deposition is a major problem in VF scarring. The molecular and macroscopic structures of type 1 collagen produced during VF healing differ from those of native collagen, which is mainly type 3. Type 3 fibers are finer than type 1 fibers and are well organized in normal VFs [56]. Deliberate VF scarring may be induced by stripping, or by electrocautery. Kumai et al. [18] demonstrated that at 1 month post-treatment, cauterized ferret VFs had white scar tissue and prominent vascularization, in contrast to normal translucent VFs. These preliminary observations of ferret VF scars were used to establish an animal model for in vivo VF scarring and injection laryngoplasty. Kumai et al. noted that the ferret is a surgically sturdy animal model that shows resilience to anesthesia. Ferrets are also easy to handle and have relatively large VFs that can be exposed and accessed easily with the same surgical instruments used to treat patients. The ferret has the advantages of being a small animal with a large larynx and relatively thick SLP layer. Therefore, injections can be targeted precisely to screen therapeutic agents for VF scars [54].

Other in vitro studies have used VF fibroblasts isolated from both animals and humans [17,18,19,56,57,58]. Myofibroblasts are largely responsible for the synthesis of type 1 collagen in wounds, wound contraction and fibrosis [59]. In 2009, Kumai et al. [18] examined fibroblasts isolated from both normal and scarred ferret VFs using light and electron microscopy. The observations indicated that the fibroblasts in the scarred ferret VFs were probably myofibroblasts, and that these myofibroblasts were involved in VF fibrosis. The study confirmed that the normal and scar tissue-derived VF fibroblasts maintained their phenotypic differences in culture, validating this in vitro scarring model. 

Recently, Kishimoto et al. [60] characterized rat VF scar fibroblasts at the transcript, transcriptome, protein and functional levels. They confirmed that this experimental in vitro model may be applicable in any suitably equipped laboratory. Branco et al. evaluated the myofibroblast profile isolated from normal and scarred human VF and suggested that VF fibroblast treated with TGFβ1 (myofibroblasts) appear to have similar phenotypic characteristics but different genotypic behavior compared to VF fibroblasts isolated from human VF scars [61]. Graupp et al. established a laryngeal fibrogenesis model employing human VF fibroblasts based on the principle of the macromolecular crowding [62]. These excellent recent in vitro VF fibroblast models would accelerate the development of the novel treatment strategy for VF scars. 

## 4. Current Treatment Options and New Injection or Implantation Materials

There is no standard modality for the prevention or treatment of VF scarring. In some VF scarring cases, significant improvements in the voice have occurred after speech therapy alone [63]. However, speech therapy alone is unlikely to result in histological improvements in VF scars. Injection laryngoplasty is currently the most common and clinically appropriate treatment for VF scarring. Among the injection materials available, steroids have proven popular due to their reputation for improving voice quality [14,15]; however, this effect remains unconfirmed. Steroids may enhance wound healing by modulating the synthesis and maturation of collagen, inhibiting fibroblast proliferation. To understand the mechanism and value of steroid delivery at the time of VF injury, Campagnolo et al. [64] evaluated histological outcomes post-injury using a rabbit in vivo model. The data supported a positive therapeutic effect of steroid, at least during the acute phase of VF injury. Recently, Mukudai et al. demonstrated in in vitro study that dexamethasone regulated TGF-β1 signaling via altered SMAD3 and SMAD7 expression which was associated with altered glucocorticoid phosphorylation. These findings provide new insight into the mechanisms of steroidal effects on VF repair [65]. Yildiz et al. demonstrated in in vivo study that the effects of estradiol or dexamethasone injections may have similar positive effects on wound healing (especially, higher elastin expression levels which is good for VF vibration) in VF injuries [66]. Further investigation of the significance of steroid injection treatment needs to be advanced. 

In addition to steroids, other materials have been injected to soften VF scars. These have included bovine collagen [67], autologous collagen [68], autologous fat [69] and HA [13,70,71]. Among these materials, HA has received widely as a new therapy for VF scarring [70,71]. As previously described, HA is considered a key molecule for maintenance of VF viscoelasticity [25]. Recently, Coppoolse et al. [72] used a rabbit model to demonstrate injecting a chemically modified HA-based scaffold at the time of VF injury could enhance wound repair and preserve viscoelasticity. Walimbe presented a review of HA and HA-based hydrogels for VF tissue engineering showing that HA is a bioactive glycosaminoglycan responsible for maintaining optimum viscoelastic properties of the VFs and hence is widely targeted in tissue engineering applications [73]. In terms of viscoelasticity, HA would be the strong candidate as scaffold for tissue engineering approach for the VF scar. 

As new candidates of injection or implantation biomaterials, Zeitels et al. [16] used polyethylene glycol (PEG) 30 as a new biomaterial implant since PEG is an established biocompatible polymer approved by the US Food and Drug Administration. Zeitels et al. demonstrated the positive effects of PEG30 hydrogel on VF structural and functional parameters without mechanically impeding the SLP. Recently, Pitman et al. demonstrated that implantation of the small intestinal submucosa as biomaterial implant on the chronic VF scar in vivo reduced the density of collagen I deposits without a negative impact or complication based on the implantation [74]. These two biomaterials would be the strong candidates for injection materials for VF scar soon in the future. Platelet-rich plasma injection to VF scar has been demonstrated as effective on improvement of wound healing using animal models in vivo [75,76]. Pitman et al. suggested that autologous transplantation of temporalis fascia into SLP for VF scar resulted in significant subjective vocal improvement that persists at least 1 year after surgery [77] and with good long-term (nearly 4 years) outcomes and high patient satisfaction [78].

However, no known materials can completely restore the disrupted LP and achieve a level of viscoelasticity identical to that of a normal VF. Therefore, to restore the pliability of VF scarring, new approaches using regenerative medicine and tissue engineering must be considered. 

## 5. New Strategies for VF Scar Including Regenerative Medicine and Tissue Engineering

**Figure 1:**
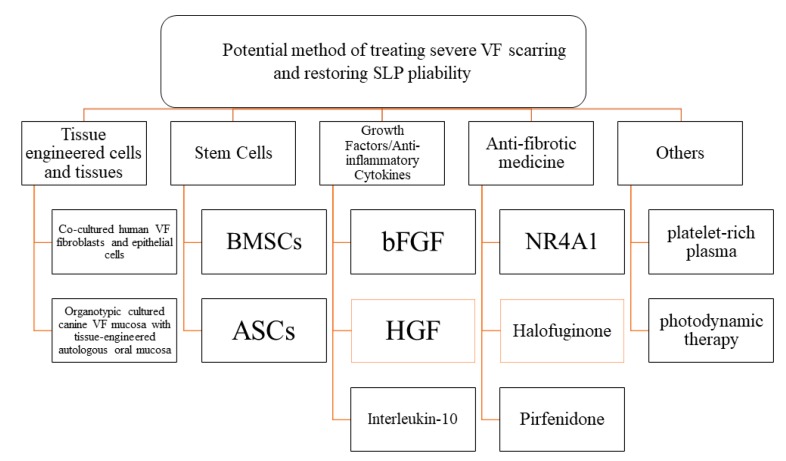
Organizational chart showing current potential treatment strategies for restoring vocal fold (VF) pliability. BMSCs, bone marrow-derived mesenchymal stromal cells; ASCs, adipose-derived stem/stromal cells; bFGF, basic fibroblast growth factor; HGF, hepatocyte growth factor; NR4A1, orphan nuclear receptor 4A1.

A new potential method of treating severe VF scarring and restoring SLP pliability involves tissue engineering, stem cells, growth factors/anti-inflammatory cytokines, antifibrotic medicine and other new technologies. (Figure 1).

### 5.1. Tissue Engineered Cells and Tissues

Although the research is at an early stage, multiple tissue engineering approaches have recently been described [12,79]. Generally, tissue engineering aims at the regeneration of biological structures with an appropriate combination of scaffolds, cells and growth factors [80]. Several cell types were selected for VF treatments, including native VF fibroblasts, autologous fibroblasts from nonlaryngeal tissues and stem cells [7]. Decellularized matrices, biological polymers and synthetic modified biopolymers were selected as candidates of scaffold [16]. Kishimoto et al. demonstrated the outcome of atelocollagen sheet (a cross-linked collagen material with abundant micropores) implantation in VF scar patients. The majority of patients exhibited gradual improvement in vocal outcome 6 months following implantation [81]. He also reviewed the scaffold research field of the VF scar and emphasized in the review paper that the atelocollagen sheet is an established clinical utility as a scaffold for dermal and epidermal repair which can be applicable for VF scars [79].

As following the principle of tissue engineering, cells, scaffolds and signaling molecules need to be properly combined for restoration of SLP pliability. Inserting suitable scaffolds by injection or three-dimensional implantation was investigated [12]. Interestingly, Tse et al. demonstrated the potential of a decellularized scaffold to serve as a tissue-engineered construct for VF replacement [82]. Overall, we still need further speculation for selecting the ideal scaffolding material, the detail process of the replacement of scaffold by selected cells and the role of growth factors in the establishment of tissue engineering approach for VF scars.

Recently, an exciting approach using bioengineered VF mucosa for voice restoration of VF scar has been reported. Welham et al. isolated human VF fibroblasts and epithelial cells and cocultured them under organotypic conditions. They demonstrated that this technique has the potential for voice restoration of VF scars [83]. Fukahori et al. presented that implantation of organotypic cultured mucosa with tissue-engineered autologous oral mucosa in the deficient VF mucosa due to resection of the VF scar could successfully restore the layered VF in vivo. The technique would be clinically advantageous for treating VF scars in the future [84].

### 5.2. Stem-Cell Therapy

Recently, researchers have used stem cells from a variety of sources in attempts to enhance healing in VFs. The two major stem-cell types being used are bone marrow-derived mesenchymal stromal cells (BMSCs) and ASCs.

#### 5.2.1. BMSCs

Kanemaru et al. firstly attempted to use BMSCs to improve regeneration of injured VF [85]. Cultured BMSCs mixed with hydrochloric atelocollagen were injected into VFs. Cells with prominent nuclei observed in the muscle tissue were identified as injected BMSCs because similar cells were not present in controls. Subsequently, mouse BMSCs were characterized and implanted into nude rat VFs [86]. These researchers concluded that tissue engineering using BMSCs may help to reconstruct VF layers by enhancing epithelial and muscle regeneration. Hertegard et al. [87] investigated the viscoelastic and histological properties of rabbit VF scars after human BMSCs were injected. Despite poor residency of the stem cells, there was some evidence that viscoelastic parameters were improved, and type 1 collagen content was reduced 1 month after the treatment [87]. Hertegard et al. also demonstrated that human mesenchymal stem cells (MSCs) injected into rabbit VFs as xenografts following the excision of chronic scars could enhance VF healing, decrease the thickness of the LP, and restore viscoelastic shear properties [88]. Ohno et al. [89] demonstrated that implanting atelocollagen sponges together with autologous BMSCs into canine VF scars significantly enhanced HA distribution and reduced dense collagen deposits within the LP, leading to better VF vibration. 

Hiwatashi et al. compared the effects of injection combined with atelocollagen to the VF scar between ASCs and BMSCs using a canine in vivo model and concluded that ASCs might have more potential to increase HA and to decrease collagen deposition in VF scars [90]. He also demonstrated that MSCs can decrease type I and III collagen levels, and suppress differentiation toward myofibroblast via blocking TGFβ1 [91]. Recently, Nagubothu et al. presented that BMSCs can accelerate the wound healing process via suppression of the inflammatory response and promotion of tissue repair in the acutely injured VF [92]. Kim et al. tried another type of MSCs derived from human nasal inferior turbinate. Interestingly, they used conditioned media derived from human nasal inferior turbinate-derived MSCs. He suggested that injection of this conditioned media into VF injury produced antifibrotic effects at least in the acute phase of injury. These effects were not far behind the effects produced by the injection of MSCs themselves [93]. Hiwatashi et al. recently presented that conditioned media derived from BMSCs alters fibroplasia in human VF fibroblasts in vitro. [94]. In the future, utility of the conditioned media derived from various types of the MSCs would provide great impact on the treatment of VF scars with provision of antifibrotic effects of MSCs for future clinical application.

#### 5.2.2. ASCs

Lee et al. evaluated the potential of ASCs to reduce scarring and atrophy in injured canine VFs. Fluorescence-labeled ASCs mixed with atelocollagen were injected into the VFs (the specific layer injected was not described). Four days later, the posterior portions of the VFs were injured using electrocautery. Many fluorescent ASCs were observed in tissue sections at 8 weeks post-injection. Hematoxylin and eosin staining also showed many immature mesenchymal cells in the subepithelial connective tissue [95]. At 24 weeks after treatment, the injected sides of the VFs showed less atrophy and fewer morphological irregularities than the control sides. Several research groups have focused on implanting ASCs. As described above, Kumai et al. [18,19] established an in vitro ferret VF scarring model and used a co-culture setup to demonstrate that ASCs cause VF-scar fibroblasts to adopt a less fibrotic profile. Hepatocyte growth factor (HGF) was one of the soluble factors which acts as an antifibrotic agent, and implanted ASCs could ameliorate VF scarring via paracrine paradigm [19] as the same with BMSCs [91,92]. Valerie et al. [96] showed that transplanting ASCs into a chronic scar could regenerate the VF by dissolving the excess collagen fibers that form the scar tissue and restoring the normal structure of elastic fibers. King et al. [97] used ASCs embedded in a hyaluronan scaffold for VF scarring. These researchers investigated the macrophage inflammatory response to allogeneic ASCs and concluded that the constructs were biocompatible. The ASCs constructs did not provoke increases in collagen expression, suggesting that grafts would be stable in the long term. Goel et al. used a rabbit VF-injury model to assess the persistence of embedded ASCs within a tissue-engineered VF mucosal replacement [98]. The tissue produced after implantation of a tissue-engineered outer VF replacement was derived from both the embedded ASCs and infiltrating native cells. These data suggested that a tissue-engineering approach may provide a well-integrated tissue graft with prolonged cellular activity and can repair severe VF scars. 

Recently, Mattei et al. [99] presented results from the first patient treated for VF scarring using ASCs implantation. The patient was a 43-year-old woman with severe dysphonia associated with VF scarring following surgical intervention to VF. Current treatment options such as medical and surgical treatments did not resolve his dysphonia. Autologous ASCs were injected locally and produced no serious adverse effects. At 1 year after surgery, the majority of the voice parameters and Voice Handicap Index had improved with almost normal voice. de Bonnecaze et al. proved that the ASCs improve healing of VF scars with morphological and functional evidence using a rabbit in vivo model. They demonstrated in the study that 6 weeks after ASCs injection, VFs presented significantly less inflammation and fibrotic response than control VFs [100]. Morisaki et al. presented that ASCs increased expressions of FGF2 and HGF and suppressed excessive collagen deposition during VF wound healing possibly in combination with upregulations of growth factors’ genes in surrounding cells in vivo [101]. Overall, these perspectives suggested that ASCs have an impact on prevention of VF scars during wound healing. 

### 5.3. Growth Factors and Anti-Inflammatory Cytokines

Over the last 10 years, Hirano et al. have been working with various growth factors to develop tissue engineering tools that can be used in regenerative medicine. Among these, basic fibroblast growth factor (bFGF) [9] and HGF [59,102,103,104,105] have shown some potential in restoring VF scars.

Hirano et al. confirmed that bFGF stimulated VF fibroblasts to produce HA and suppressed the production of type 1 collagen, inhibiting scar formation [9]. In a trial involving 15 patients with VF scars, Hirano et al. showed that bFGF improved voice parameters especially when combined with a surgical approach [9]. Recently, Hiwatashi et al. presented that collagen-gelatin sponge and bFGF combination therapy may have therapeutic potential for treating VF scars [106]. Additionally, Suzuki et al. from the same group demonstrated that local application of bFGF during VF wound healing has the potential to prevent VF scars. Therefore, injection of bFGF to injured portion of VF at the time of phonomicrosurgery would prevent postoperative scar formation [107]. Ban et al. demonstrated the positive effect of bFGF especially for chronic VF scars with an in vivo animal study and prospective human clinical trial [108].

Hirano et al. also used HGF as an alternative treatment for VF scarring due to its antifibrotic properties. HGF is involved in embryogenesis, angiogenesis, and tissue regeneration, and HGF administration has been used to prevent or resolve liver, kidney and lung fibrosis in animal models [103]. Moreover, like bFGF, HGF stimulates HA production and suppresses type 1 collagen production by both canine and human VF fibroblasts in vitro [103,104]. Hirano et al. demonstrated the prophylactic effect of HGF on VF scar using a rabbit in vivo model [59]. This study suggested that HGF may help to prevent or reduce scarring in injured VFs. Together, these studies suggest that HGF has therapeutic potential for preventing and treating VF scarring. Furthermore, a drug delivery system may prolong the effect of locally applied HGF [10]. The best method for administering HGF with an improved drug delivery system should be determined in future studies. 

Hirano et al. [106] recently described impressive clinical trial results for HGF. This phase I/II, first-in-human clinical trial involved injections of a recombinant dHGF drug, KP-100LI, into VF scars in 18 patients. No safety concerns were uncovered, and there were significant improvements in the Voice Handicap Index-10, VF vibratory amplitude, and GRBAS (grade, roughness, breathiness, asthenia, strain) scale among the 18 patients.

The role of fibroblasts as inflammatory mediators are known to express a rich source of inflammatory cytokines, chemokines and lipid mediators with VF injury. King et al. demonstrated that fibroblasts derived from scar, polyp and normal VF tissue co-cultured with macrophages can modulate their paracrine signaling during early cytokine expression (i.e., tumor necrosis factor-α (TNF-α), Interleukin-10 (IL-10), IL-12) and subsequent chemokine and growth factor expression (i.e., IL-6, IL-8, Monocyte Chemotactic Protein-1(MCP-1), transforming growth factor-β (TGF-β)). Especially, VF scar fibroblast stimulated high IL-10 and low IL-12 expression from activated macrophages [58]. 

Recently, Chen et al. demonstrated that a decrease in pro-inflammatory markers (TNF-α, IL-6) and an increase in anti-inflammatory markers (i.e., IL-10/IL-12) may favor antifibrotic outcomes via reduction in fibrotic proteins and supported the therapeutic potential of HGF and IL-10 for VF scar treatment using in vitro models [109]. These perspectives would bring the light of hope on future strategies targeting specific activated macrophages phenotypes for establishing an anti-inflammatory approach for the VF scar.

### 5.4. Antifibrotic Medicine 

Recently, several candidates of the antifibrotic agents for VF scar treatment have been investigated. Zhou et al. presented that prostaglandin E2 exhibits an antifibrotic effect on the VF fibroblasts in vitro and this would be one of the candidates of antifibrotic agents for VF scar treatment [110]. Hiwatashi et al. described orphan nuclear receptor 4A1 (NR4A1) is an endogenous inhibitor of TGF-β-induced VF fibrosis. Therefore, cytosporone-B, as an NR4A1 agonist, may be a new therapeutic candidate for VF scar [111]. Another candidate of antifibrotic agent for VF scars, Halofuginone, an inhibitor of the TGF-β fibrotic cascade selectively inhibits smad-3 activation of pro-collagen mRNA transcription. This process suppressed type 1A collagen production with minimal effect on other normal collagen subtypes. Allen et al. demonstrated that administration of halofuginone resulted in decreased elastin and collagen deposition in acute VF injury using ovine in vivo model [112]. 

Drug repurposing has recently become a popular research strategy for developing various types of “new” medicines [113,114,115,116,117]. This involves known drugs being used to treat different diseases. A major advantage of drug repurposing is that it avoids the high costs associated with developing entirely new drugs. Our group has focused on PFD (5-methyl-1-phenyl-2-[1H]-pyridone), which has a combination of anti-inflammatory and antifibrotic effects. PFD acts by regulating the TNF-α and TGF-β1 pathways, as well as by modulating cellular oxidation [118]. Since the late 1990s, studies have demonstrated that PFD can reduce fibroblast proliferation and collagen synthesis and deposition, both in vitro and in patients [118,119]. Idiopathic pulmonary fibrosis (IPF) is a rare progressive unknown pathology of lung fibrosis characterized by deterioration of lung function [120]. Initial open-label trials presented that PFD could be used to treat IPF [121], and the first prospective clinical studies demonstrated that PFD slowed the deterioration of lung function, [122]. Based on these findings, in 2008, the Japanese Ministry of Health, Labor, and Welfare approved the use of PFD to treat mild-to-moderate IPF, which suggested PFD can be clinically applicable for progressive pathology of fibrosis in other organs, such as VF scars. TGF-β1 is one of the most extensively studied profibrotic cytokines. We hypothesized that PFD would suppress TGF-β1 activity, as described previously [123,124]. This would reduce both collagen production and collagen gel contraction in vitro. Kumai et al. [17] proved in vitro the antifibrotic effects of PFD on fibroblasts isolated from ferret VF scars. They demonstrated that PFD treatment significantly decreased mRNA expression of collagen type 1, significantly increased mRNA expression of TGF-β1, and significantly suppressed collagen gel contraction. However, PFD had no significant effect on the expression of α-smooth muscle actin (α-SMA). These data from our study suggest that PFD suppresses the translocation of p-Smad2/3 from the cytoplasm to the nucleus. Interestingly, the expression of hyaluronan synthase 2 mRNA, which induces HA production, was significantly increased in the presence of PFD. As described previously [25,26], HA is crucial for VF pliability, therefore our preliminary results demonstrate that PFD can help to restore VF pliability in vitro. Additionally, recently, we have confirmed that PFD has antifibrotic effects in vivo using a ferret model (unpublished data). In addition, the level of collagen type 1, which is the principal component of VF scars produced by electrocauterization, was reduced by injecting PFD into ferret VF scar. This may be because the α-SMA level was also reduced, reflecting decreased activation of myofibroblasts. Overall, injecting PFD into VF scars is a promising novel treatment that may be clinically applied in the near future.

### 5.5. Gene Therapy

Branski et al. described Smad3 as a key biochemical signaling protein which regulates fibrotic phenotype and a potential target for siRNA-based therapeutics [125,126]. Smad3 is critical to TGF-β signaling which is fundamental to wound healing in the VFs and other tissues. Recently, Karaja et al. demonstrated that lipitoid which was initially developed for intracellular plasmid DNA delivery, effectively knocked down Smad3 expression across multiple transfection conditions [127]. Moreover, Hiwatashi et al. [128] from the same group demonstrated firstly the targeted gene manipulation in the VFs as well as the potential utility of lipitoid for localized delivery of genetic material in vivo. Several recent reviews demonstrated the strong impact of the gene therapy on hypertrophic scars [129] and liver firbosis [130]. The concept presented here needs to be applied for the development of the innovative gene therapy for the VF scar in the future.

### 5.6. Laser Therapy and Cryotherapy

Shue et al. attempted to provide mechanistic insight into the clinical utility of angiolytic lasers for VF scars. As the same with previously obtained perspectives, it appears that the positive effect of angiolytic lasers are mediated by matrix metalloproteinase. The authors demonstrated that potassium-titanyl-phosphate (KTP) laser has the potential to augment wound healing in a rat VF injury model and utility of KTP laser may serve as a therapeutic tool for the management of VF fibrosis [131]. Recently, Zhang et al. demonstrated that KTP laser and Yttrium-Aluminum-Garnet (Nd:YAG) laser had positive effect in VF scars. These positive effects may be through the down-regulation of the TGF-β pathway and the reduced production of inflammatory mediators, and further reducing collagen secretion and deposition [132]. Luo et al. also recently presented that low-level laser therapy can stimulate the proliferation and migration of human VF epithelial cells in culture as well as increase the expression of some genes involved in the tissue healing process [133]. From the same group, Gong et al. recently demonstrated that cryotherapy which is the localized application of extremely cold temperatures for eliminating lesions or relieving physical suffering was effective to minimize VF scarring especially administered at the time of injury [134]. Zhang et al. recently demonstrated that photodynamic therapy induces antifibrotic alterations in primary human vocal fold fibroblasts in vitro [135]. Overall, these new technological approaches would bring drastic progress in the treatment for VF scars soon in the future.

## 6. Current Obstacles to Restoring SLP Pliability

**Figure 2:**
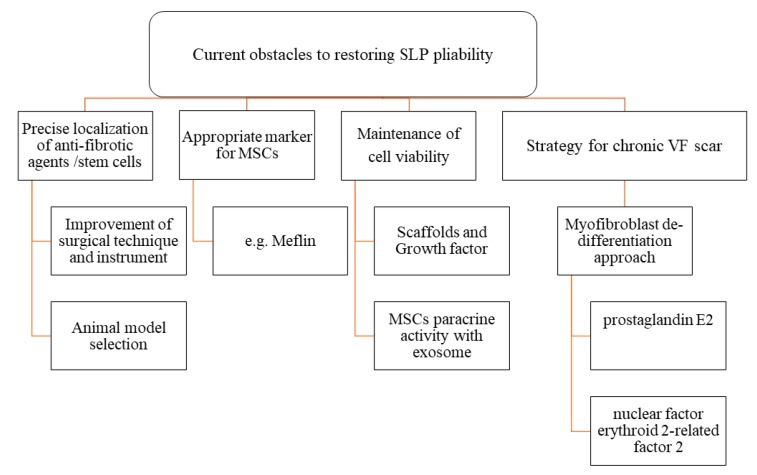
Organizational chart showing current obstacles to restoring VF pliability.

An organizational chart showing current obstacles to restoring VF pliability is described in Figure 2.

### 6.1. Precise Targeting of Antifibrotic Agents and Stem Cells

It is difficult to precisely inject small animals such as rats and rabbits. This is probably why cells that are targeted to the LP are often found in the VF muscles. Kumai et al. showed that the SLP in some of these animal models is not much thicker than the diameter of the needles used for the injections [54]. However, using smaller needles to increase precision could damage the injected stem cells. Animal selection in terms of the width of the SLP layer as injection target is the key for overcoming this issue. As mentioned before, the ferret has the advantages of being a small animal with a large larynx and relatively thick SLP layer. Therefore, injections can be targeted precisely to screen therapeutic agents for VF scars [54]. 

### 6.2. Appropriate Markers to Distinguish Normal Fibroblasts from Stem Cells

Identifying stem cells that have differentiated to become fibroblasts is also problematic, because fibroblasts and stem cells presents similar patterns of marker expression (positive for vimentin, CD44, and CD90; negative for epithelial-cadherin, leukocyte-CD45 and endothelial-CD31) [136]. Therefore, the number of stem cells that have differentiated into normal fibroblasts might be underestimated in some studies. Clearly, there is a need for appropriate markers to distinguish normal fibroblasts from stem cells. 

Recently, Maeda et al. demonstrated that Meflin is a potential specific marker for cultured mesenchymal stem cells (MSCs) and their source cells in vivo. This kind of specific marker for MSCs would be helpful for accurate evaluation of MSCs implantation approach for VF scars in the future [137]. Although, not for VF scar treatment, Voss et al. recently demonstrated that histone variants are useful as ASCs biomarkers for long-term injection medialization laryngoplasty [138]. These markers would be helpful for the accurate identification of the stem cell differentiation process in the stem cell implantation approach for VF scar.

### 6.3. Maintenance of Cell Viability and Its Antifibrotic Effect on VF Scars via Paracrine Paradigm

As stem cell viability in the acutely injured VFs was low in many previous studies, the mechanisms by which stem cells improve VF pliability remain uncertain. Studies published to date show that relatively few non-autologous stem cells (0.2–5%) survived at 1 month after injection [85,86,87,88,89,139]. In addition, there is little evidence that injected stem cells can differentiate into VF fibroblasts, which are responsible for synthesizing the SLP ECM. However, previous stem-cell studies have demonstrated the improvement of the VF viscoelasticity after stem cell injections [86,87,88,140]. Therefore, we need to confirm that the cells are sufficiently viable before we can discuss the antifibrotic effects of stem cells in animal models. 

Baglio et al. mentioned in the review of the therapeutic potential of MSCs that the utility of MSCs for tissue regeneration was initially based on the hypothesis that MSCs would differentiate into specific local cells within the damaged tissue. However, it now appears that only a small proportion of implanted MSCs actually survive in host tissues. Thus, the predominant mechanism by which MSCs contribute to tissue regeneration seems to be through their paracrine activity with exosome [140]. Kumai et al. noted that ASCs had paracrine antifibrotic effects on fibroblast function [18,19]. For example, scar fibroblasts co-cultured with ASCs produced less collagen and proliferated less rapidly. These observations are consistent with previous studies demonstrating that stem cells can offer therapeutic effects via paracrine activity. For example, Parekkedan et al. [141] studied interactions between BMSCs and activated hepatic stellate cells which mainly relate to matrix deposition in liver fibrosis. The BMSCs reduced collagen secretion and increased apoptosis in the stellate cells via soluble factors such as IL-10, TNF-α (the key molecules inhibiting matrix deposition), and HGF which would induce apoptosis [142]. In the paracrine paradigm, ASCs interact with fibroblasts isolated from fiboris and stimulate them, via cytokines, to decrease collagen production and proliferation, consequently, fibrosis can be reduced. Previous studies showed that BMSCs can reduce fibrosis in the liver and myocardium, and that cytokines produced by the stem cells are responsible for these reductions [143,144]. In other organs, including the lungs and kidneys, the beneficial effects of BMSC-based therapy, such as angiogenesis, anti-inflammation, and anti-apoptosis, are largely mediated via paracrine paradigm, rather than by differentiation of BMSCs into local cell types [145]. 

Recently, Ti D. et al. demonstrated that MSCs may release plenty of exosomes which are able to regulate and regenerate the balance of macrophages with the resolution of chronic inflammation after lipopoly-saccharide treatment [146]. Based on these exciting recent perspectives, ideally, these MSCs-mediated effects would be helpful for especially the resolution of chronic inflammation in the VF scar via stem cell implantation approach. 

### 6.4. Strategies for Chronic VF Scarring

One important issue is whether tissue engineering approaches would be effective for treating chronic VF scars. Although tissue engineering is sure to be useful for preventive treatments during surgery, there is probably a greater clinical need to treat established scars. To study chronic VF scarring in animal models, additional time is needed for scar formation prior to treatment. Furthermore, certain cytokines and growth factors in the scar microenvironment may affect the survival of antifibrotic agents and the proliferation or differentiation of stem cells. 

Currently, a powerful new approach to successfully resolve chronic scarring is being developed. This procedure involves using prostaglandin E2 and nuclear factor erythroid 2-related factor 2 nuclear translocation to de-differentiate myofibroblasts into normal fibroblasts [147,148,149]. As mentioned before, Zhou et al. already presented that prostaglandin E2 exhibits an antifibrotic effect on the VF fibroblasts [110]. These perspectives would be helpful for establishing strategies for chronic VF scarring in the future.

## 7. Conclusions

Overall, various promising and encouraging strategies for restoring the pliability of the VF scar are currently being developed, however, are mid-way as is the situation so far. Despite basic and clinical otolaryngological research to develop novel treatments, VF scarring remains difficult to resolve for two major reasons. First, to restore pliability, antifibrotic agents or cells must be precisely targeted to, and remain viable in, the SLP. In this regard, amelioration of VF scarring via the paracrine paradigm using MSCs needs to be investigated further. Second, a basic strategy for regenerating chronic VF scars is needed. In addition to preventing scar maturation, potential methods for transforming chronic VF scar tissue into normal pliable VF tissue need to be investigated.
